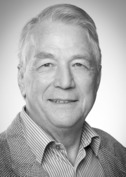# In Search of Bioactivity – Phyllobilins, an Unexplored Class of Abundant Heterocyclic Plant Metabolites from Breakdown of Chlorophyll

**DOI:** 10.1002/ijch.201900012

**Published:** 2019-04-24

**Authors:** Simone Moser, Bernhard Kräutler

**Affiliations:** ^1^ Pharmaceutical Biology, Pharmacy Department Ludwig-Maximilians University of Munich Butenandtstraße 5–13 81377 Munich Germany; ^2^ Institute of Organic Chemistry and Centre of Molecular Biosciences University of Innsbruck. Innrain 80/82 6020 Innsbruck Austria

**Keywords:** antioxidants, N-heterocycles, natural products, porphyrin(oid)s, tetrapyrroles

## Abstract

The fate of the green plant pigment chlorophyll (Chl) in de‐greening leaves has long been a fascinating biological puzzle. In the course of the last three decades, various bilin‐type products of Chl breakdown have been identified, named phyllobilins (PBs). Considered ‘mere’ leftovers of a controlled biological Chl detoxification originally, the quest for finding relevant bioactivities of the PBs has become a new paradigm. Indeed, the PBs are abundant in senescent leaves, in ripe fruit and in some vegetables, and they display an exciting array of diverse heterocyclic structures. This review outlines briefly which types of Chl breakdown products occur in higher plants, describes basics of their bio‐relevant structural and chemical properties and gives suggestions as to ‘why’ the plants produce vast amounts of uniquely ‘decorated’ heterocyclic compounds. Clearly, it is worthwhile to consider crucial metabolic roles of PBs in plants, which may have practical consequences in agriculture and horticulture. However, PBs are also part of our plant‐based nutrition and their physiological and pharmacological effects in humans are of interest, as well.

## Introduction

Breakdown of chlorophyll (Chl) is a visual process that degrades an estimated amount of roughly 1000 million tons of Chl, annually, on Earth^1^ and furnishes the corresponding amount of bilin‐type linear tetrapyrroles,^2^ named phyllobilins (PBs).^3^ Chl breakdown has been studied most extensively in senescent leaves,^4^ in which colorless PBs accumulate as seemingly ‘useless’ products of a tightly controlled mere Chl detoxification process.^5^ Structure elucidation of PBs has prompted a change of paradigm eventually:4c,4g,^6^ due to the bilin‐type build‐up of their core structures and their specific further decoration with (glycosidic) functional groups, PBs are considered, nowadays, to represent complex heterocyclic natural products with structures of significant pharmaceutical interest,4j,^7^ and with a vast potential for physiological activity in plants.4g,4j

**Scheme 1:**
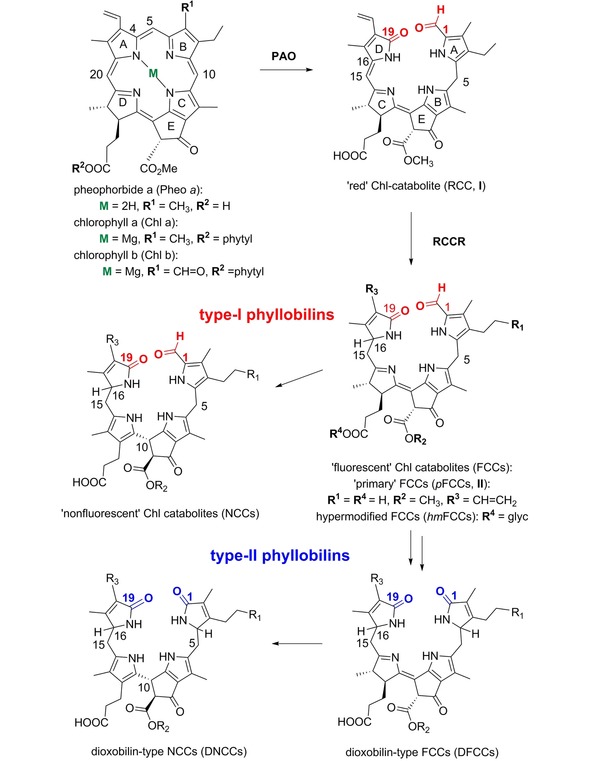
Structural outline of the PAO/phyllobilin pathway of Chl breakdown in higher plants. Oxygenolytic opening of the porphyrinoid macro‐ring of Pheide a generates 1‐formyl‐19‐oxobilins (type‐I phyllobilins) first, from which a second set of colorless PBs branches off, the 1,19‐dioxobilin‐type or type‐II PBs.

In senescent leaves4b,4d,4f,^8^ and in ripening fruit6a,^9^ tightly regulated enzymatic processes degrade Chls *a* and *b* to pheophorbide *a* (Pheide *a*)4b,4d,^10^ (see Scheme 1). In an oxygen dependent process, catalyzed by Pheide *a* oxygenase (PAO),^11^ Pheide *a* is then ring‐opened at its ‘northern’ meso‐position.^12^ This furnishes a cryptic 1‐formyl‐19‐oxo‐bilin type tetrapyrrole,^13^ the red Chl catabolite (RCC, **I**),^14^ which is the common progenitor of the PBs.4b,4g,^10^ The site of the oxygenolytic opening of the porphyrinoid macroring of Pheide *a* (to RCC **I**) corresponds in a striking way to that of heme opening by heme oxygenase (HO).^15^ This ‘heme‐suicidal’ protein cleaves the porphyrinoid macroring of heme between its rings A and B, removing the heme meso‐carbon as carbon monoxide and furnishing biliverdine, the biosynthetic precursor of the other bilins (see below, Scheme 3).^15^ In contrast to HO, the mono‐oxygenase PAO does not cut out the meso‐carbon of its substrate (Pheide *a*) and generates a 1‐formyl‐19‐oxo‐bilin type Chl catabolite.^12^

RCC **I** is reduced by RCC reductases (RCCRs)^16^ in a highly stereo‐selective plant specific way to one of two C16‐epimeric forms of the colorless ‘primary’ fluorescent Chl catabolites (*p*FCCs, **II**).^17^ The C16‐epimeric *p*FCCs are either hydroxylated at their C3^2^ position (to produce 3^2^‐OH‐*p*FCCs,^18^ also named ‘secondary’ FCCs or *s*FCCs), or directly exit the chloroplast to arrive in the cytosol, where FCCs undergo further enzyme catalyzed modifications.4c,4d An important FCC‐core modification is the oxidative removal of the formyl group,^19^ producing a 1,19‐dioxobilin‐type FCC, also named DFCC,^20^ and opening up the branch of the dioxobilin‐type PBs.4c,4g As 1‐formyl‐19‐oxobilin‐type PBs are the ones generated first from Chls, they are also classified as type‐I PBs.4g The 1,19‐dioxobilin‐type PBs, which are formed subsequently from their FCC precursors, are correspondingly grouped as type‐II PBs.4g,4j Typical FCCs and DFCCs are, both, short lived and prone to a highly stereo‐selective isomerization to the corresponding ‘non‐fluorescent’ Chl catabolites (NCCs^21^ or DNCCs^20^), also classified as phyllobilanes (Scheme 1).

## Phyllobilins Display a Rich Structural Variety

**Scheme 2:**
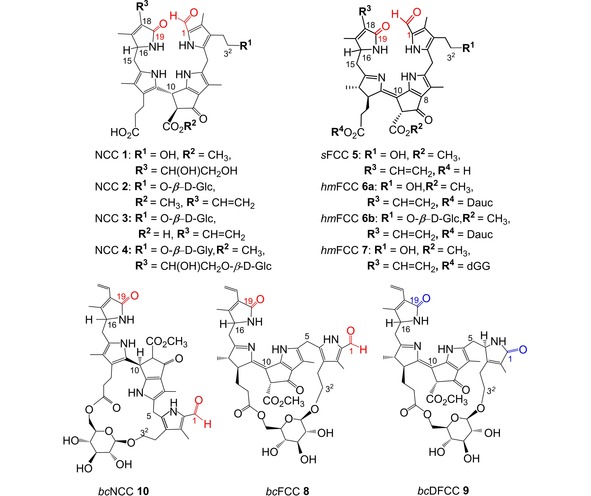
Chemical formulae of representative natural phyllobilins. *Top*: Formulae of the NCCs **1–4** and of FCCs, *s*FCC **5** and *hm*FCCs **6** and**7** (Glc=D‐glucopyranosyl; Dauc=4’‐daucyl; dGG=6‐α‐galactopyranosyl‐(1‐6)‐β‐galactopyranosyl‐(1‐1)‐glyceryl). *Bottom*: Formulae of the three known bicycloglycosidic PBs, *bc*NCC **10**, *bc*FCC **8** and *bc*DFCC **9**.

A colorless non‐fluorescent phyllobilane, or (phenomenologically) phylloleucobilin (PleB), the ‘non‐fluorescent’ Chl catabolite *Hv*‐NCC‐1 (**1**) from senescent leaves of barley (*Hordeum vulgare*), was the first non‐green Chl catabolite to be identified unambiguously.^8^,^22^ NCCs fall into two C16‐epimeric classes, either with the ‘normal’ or ‘n‐type’ or of the ‘epimeric’ or ‘epi‐type’ C16 configuration, as do their (often elusive) FCC precursors. Similar to *Hv‐*NCC‐1 (**1**), the natural NCCs carry a variety of peripheral functional groups.4g Typical peripheral NCC modifications are *β*‐D‐glucosyl and/or malonyl units decorating the OH‐group at C3^2^,^23^ dihydroxylation at the C18‐vinyl group,^8^ as well as hydrolysis of the methyl ester group at C8^2^,^23^ and their various combinations,4e,4g,^24^ such as found in the NCCs **2** and **3** from senescent leaves of tobacco^25^ and from oilseed rape,^23^ respectively. The 3^2^, 18^2^‐di‐*β*‐D‐glucosyl‐NCC **4** has been identified in senescent leaves of plum trees (see Scheme 2).^26^

Natural NCCs have been deduced to be isomers of the corresponding natural FCCs, or phyllolumibilins (PluBs) which only exist fleetingly and most of which are still elusive.4c,4g An exception from this latter ‘rule’ are *s*FCCs, such as 3^2^‐OH‐*epi‐p*FCC (**5**), which has been isolated from senescent leaves of banana plants,6c and which represents the typical precursor of a range of (still elusive) FCCs carrying further modifications.4g A second exception concerns the so called ‘hypermodified’ FCCs (*hm*FCCs), in which the propionate side chain is esterified.4g,6a *hm*FCC esters, such as the banana FCCs **6 a** and **6 b**, are persistent and they do not isomerize easily to corresponding NCCs.6a,^27^ As was discovered with ripe (yellow) bananas, *hm*FCCs are a source of blue luminescence in the peel of intact yellow bananas.6a In the propionate ester function of the predominant FCC **7** in senescent banana leaves the polar head group of the major chloroplast membrane lipid is found, a 6‐α‐galactopyranosyl‐(1‐6)‐β‐galactopyranosyl‐(1‐1)‐glyceryl (dGG) unit.4h

In a further unique group of *hm*FCCs the (glycosidic) propionate ester function is attached a second time, at C3^2^, furnishing well‐structured bicycloglycosidic PBs (*bc*PBs), and making such *bc*FCCs persistent, as well.4j In fact, in the extracts of senescent leaves of the grapevine Chardonnay not only the *bc*FCC **8** was detected, but also the corresponding, stable *bc*DFCC **9**.4j The first *bc*PB discovered was actually the *bc*NCC **10**, which was found in senescent leaves of the Wych elm tree (see Scheme 2).^7^ For calculated structures of the *bc*PBs **8**–**10**, see [4j,7]. The *bc*PBs are first examples of (tetra)‐pyrrolic natural products with bicycloglycosidic structures, reminding of related bicyclic sugar‐derivatives that have a range of interesting bioactivities, e. g., on cellular growth (cancer)^28^ and as antibiotics.^29^

**Scheme 3:**
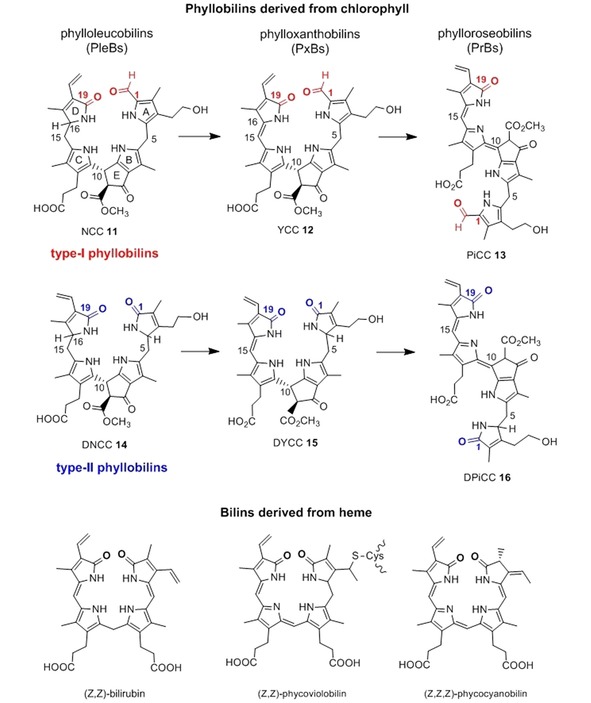
Phyllobilins from degradation of Chl and bilins from heme are remarkably related structurally. *Top*. Chemical formulae of a type‐I phyllobilane (NCC) and of the corresponding phyllochromobilins, the YCC **12** and the PiCC **13**. *Middle*. Chemical formulae of a type‐II phyllobilane (DNCC) and of the corresponding type‐II phyllochromobilins, the DYCC **15** and the DPiCC **16**. *Bottom*. Chemical formulae of the heme‐derived bilins bilirubin, of (protein ligated) phycoviolobilin and phycocyanobilin (all with Z‐configuration at their unsaturated meso‐positions).

The colorless and non‐fluorescent phyllobilanes, or phylloleucobilins (PleBs), such as the NCC **11** from fall leaves of the deciduous Katsura tree (*Cercidiphyllum japonicum*), have been found accumulating in some senescent leaves in amounts corresponding to the Chls present in the corresponding green leaves.4f In many cases, however, the PleBs recovered corresponded to only 10–30 % of that amount, raising the question of the further fate of the Chl catabolites in the intact senescent leaves,^8^,^30^ suggesting decomposition of PleBs or their removal from their original environment by extracellular transport. Indeed, in support of the former possibility in the senescent leaves, yellow and pink colored Chl catabolites have been identified4i,^31^ that arise via oxidation of the first formed phyllobilanes by a still hardly characterized ‘oxidative activity’^32^ that is frequently found in leaves, as is evident by the occurrence of the products of the oxidation reaction, the PxBs. The phylloxanthobilin (PxB) **12** was the first detected yellow Chl catabolite (YCC),31a in which a π‐conjugated chromophore extends over the two ‘Western’ rings C and D and is structured like the one of bilirubin.^33^ In the absence of light, the PxB **12** is generated in leaf extracts in the 15*Z*‐form exclusively (but isomerizes to the *E*‐form by light).31a,^32^ The PxB **12** is easily oxidized further, furnishing the pink colored Chl catabolite (PiCC) **13**, the first known example of a natural phylloroseobilin (PrB).31b,^34^ The unsaturated π‐conjugated system of the PrB **13** now stretches over rings B to D and features *E*‐configuration at the unsaturated C10 meso position. X‐ray crystal structures of the PxB **12** and of the PrB **13**, confirmed their basic structures, which were first derived by NMR‐spectroscopic means (see^35^ and^34^, respectively). The chromophore of **13** reminds of a red‐violet heme‐derived bilin of plants, named phycoviolobilin.33b A related set of PBs, as represented by the type‐I phyllochromobilins **12** and **13**, is available from oxidation of DNCCs, the type‐II phyllobilanes. Hence, oxidation of the DNCC **14** by the ubiquitous ‘oxidative activity’ furnished the DYCC **15**,^36^ a proto‐type‐II yellow PB, which is readily oxidized further in the presence of air to the corresponding type‐II PrB, the DPiCC **16** 37 (see Scheme 3).

The conversion of the polar phylloleucobilins to phylloxanthobilins and phylloroseobilins is difficult to rationalize by being a mere detoxification process. After the biological investment of the breakdown pathway to achieve de‐conjugation of the π‐system and higher water solubility of the catabolites, these phyllochromobilins are again more apolar and feature extended π‐systems resulting in interesting chemical and physical properties (see below) – intensifying the question about the biological importance of the phyllobilins. Hence, a look at the bio‐relevant chemical properties of the phyllobilins is very worthwhile.

### Bio‐Relevant Chemical Properties of Phyllobilins

**Scheme 4:**
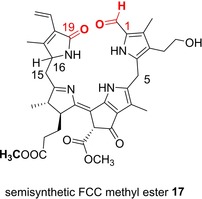
Structural formula of the semisynthetic FC methyl ester **17**

As their classification suggests, FCCs or type‐I phyllolumibilins (PluBs), were first identified provisionally by their strong blue fluorescence,4a which exhibits an emission maximum near 450 nm.^38^ Furthermore, the emission spectra exhibited by the persistent natural *hm*FCCs,6a,^39^ as well as by the semisynthetic FCC methyl ester **17** (see Scheme 4),^38^ were remarkably characteristic of intact ripe bananas6a,^39^ and of senescent yellow leaves of the banana plant.4h The blue fluorescence of ripe, yellow bananas and of the leaves of banana plants, originating predominantly from natural persistent *hm*FCCs, was proposed to be a signal to fruit eating animals, interested in harvesting and eating the ripe banana fruit.4h,6a Investigations with the FCC methyl ester **17** furnished data on the fluorescence quantum yield (0.21).^38^ Further photo‐physical studies with the FCC methyl ester **17** revealed it to be an excellent sensitizer for the formation of singlet oxygen (^1^O_2_)^38^ with a correspondingly high quantum yield for ^1^O_2_‐formation of about 0.6. Hence, natural FCCs are seemingly unprecedented cytosol‐based photo‐sensitizers for ^1^O_2_, hence, producing a short lived signal in plant cells that may, e. g., help induce stress response.^40^

**Scheme 5:**
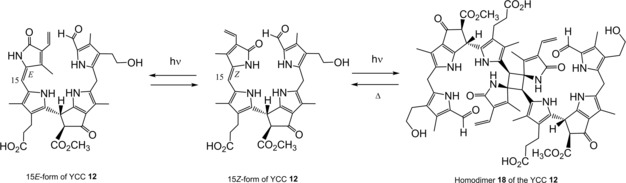
Photo‐induced reactions of the YCC **12**. *Z*/*E* photo‐isomerization (left) and photo‐induced dimerization of by [2+2]‐cycloaddition (right).

The bright yellow and pink colors of some tetrapyrrolic pigments, such as the PxBs **12** and **15**, as well as the PrBs **13** and **16**, may contribute to the fall colors of senescent leaves,4g,4i,^31^ as well as to the colors of ripe fruit.9c The structures of typical isolation forms of the natural PxBs display *Z*‐configuration of the C15=C16 double bond. However, as is typical of heme‐derived bilins, absorption of light energy induces cis‐trans isomerization of specific bonds of the chromophores of phyllochromobilins. In extracts of fall leaves of *C. japonicum*, not only the NCC **11**, but also YCC **12**, its oxidation product was detected (both, the 15*Z* and its 15*E* isomer forms of **12** were found in the leaf extracts). The (15*Z*‐isomers of the) PxBs, indeed, undergo reversible light‐induced isomerization to the corresponding less stable 15*E*‐isomers (see Scheme 5). However, typical type‐I PxBs feature a strikingly medium‐dependent photochemistry in their 15*Z*‐form. When dissolved in less polar solvents or in membrane mimetic detergent solutions the YCC **12*Z*** undergoes a clean photo‐induced [2+2]‐cycloaddition reaction at its C15=C16 double bond, to furnish the unstable, strained C2‐symmetric homodimer 1**8** (Scheme 5).^35^ The type‐II PxB **15** also undergoes the photo‐induced 15*Z*/15*E* isomerization but, in contrast to **12**, **15** does not dimerize by [2+2]‐cycloaddition at its C15=C16 double bond.^36^

**Scheme 6:**
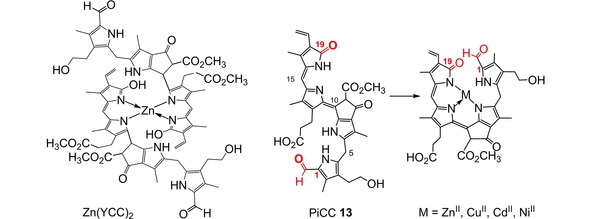
Phyllochromobilins bind transition metals effectively. Binding of various transition metal ions by the tridentate PiCC **13** (in 1 : 1 complexes, right) and of Zn(II)‐ions in a 1 : 2 complex by the bidentate YCC **12** (left).

Phyllobilanes (NCCs and DNCCs) display no capacity for coordination of (transition) metal ions.^41^ In contrast, their more unsaturated oxidation forms, the corresponding type‐I phyllochromobilins, such as YCCs and PiCCs, bind transition metals very effectively.^41^ Hence, the (hardly luminescent 15Z‐form of the) PxB **12** behaved as a bidentate ligand for zinc‐ions, furnishing an orange‐yellow 2 : 1 Zn(II)‐complex (Scheme 6) that emitted green fluorescence.^42^ The weakly luminescent PrB **13** behaved similarly, and behaved as a tridentate ligand for a variety of divalent transition metal ions.^34^ Such metal ions were bound in blue colored metal complexes after conversion of **13** from its stable 10*E*,15*Z*‐arrrangement into its (instable and elusive) 10*Z*,15*Z*‐form. The complexes of **13** with the closed shell transition metal ions Zn(II) and Cd(II) were strongly red fluorescent, signaling the presence of such transition metal ions down beyond the nM range (Scheme 6).^34^ Preliminary investigations with the DPiCC **16** have indicated a similar capacity for metal complexation,^37^ as is known for the PiCCs.^34^ Bi‐ or tridentate binding of transition metal ions by phyllochromobilins modifies their reactivity.^41^ Such binding of transition metal ions may be useful for the plant^41^ by producing toxins against pathogens,^43^ by providing a new source of photo‐generated ^1^O_2_,^44^ or by playing a role in heavy metal transport and detoxification.^45^

## Beyond Chlorophyll Detoxification – Potential Bioactivities of Phyllobilins

### Phyllobilins in Fruit and Vegetables

**Figure 1:**
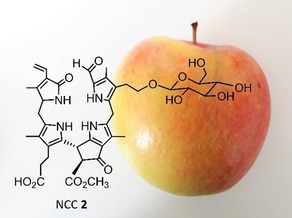
Phyllobilins are part of human nutrition. Structure of NCC **2**, a phylloleucobilin that occurs in the peels of ripe apples and ripe pears.9a

Chlorophyll breakdown furnishes various phyllobilins (PBs) in plants, not only during senescence,4d but also during the ripening process.9a,^46^ Vegetables and fruit make up a substantial part of human diet; as a consequence, we ingest PBs every day. The amount that one takes up depends on nutrition habits, and quantification of PBs in different food has so far lagged behind. Interestingly, the widespread availability of the PBs in our plant based nutrition has hardly been noticed. Nevertheless, Müller et al detected phyllobilins in the peels of ripe apples and pears and determined their amounts as about 0.6 μg of phylloleucobilins present in one square centimeter of the peel (Figure 1).9a

In terms of PB structures, the peels of apples and pears are ‘the same’ – each contain two type‐I phylloleucobilins, NCC **2** and NCC **11**; the peels of ripe pears, however, contained considerably higher quantities of NCCs compared to the peels of ripe apples.9a Recent advances in analytical methods tailored towards PBs have facilitated the detection of such natural products.4e,^24^ In recent years, a range of fruit and vegetables were investigated in terms of their PB contents; as a consequence, various NCCs, DNCCs and YCCs were detected in apricots and plums,9c,^26^,^30^ loquat fruit,9b lemons,^47^ olives,^48^ spinach,^49^ and broccoli;^50^ it is anticipated that many more are to follow.

**Figure 2:**
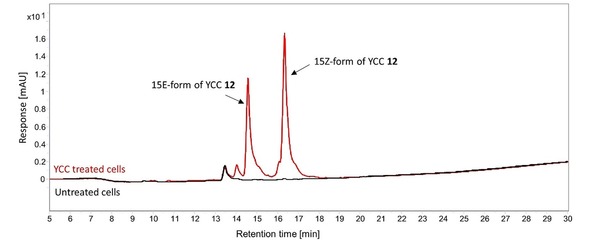
The PxB **12** is taken up by HEK‐293 cells, as shown by HPLC analyses of lysates of PxB treated cells (red trace) versus an untreated control (black trace), detection at 420 nm. For this assay, YCC **12** was prepared following the published procedure. 31a 2 million HEK‐293 cells were seeded in a 100 mm Petri dish one day prior to the treatment with 80 μM YCC **12**. After 5 hours, cells were scraped off, washed twice with PBS buffer (phosphate buffered saline 10 mM pH 7.4) and lysed with 50 % acetonitrile in PBS buffer for 1 h on ice. The lysate was clarified by centrifugation and directly analyzed by HPLC.^52^

A diet rich in plant based food would therefore lead to the ingestion of considerable amounts of PBs. The fate of the PBs in the human body, however, is unknown. Metabolization studies and LADME (liberation, absorption, distribution, metabolism, excretion) data is so far missing for the PBs. First exploratory in vitro studies on digestive stability of PleBs from ripe pepper fruit look promising, as does the evidence for uptake of a PleB (named *Ca*‐NCC‐1) by human intestinal cells (CaCo‐2 cell monolayers).^51^ Our own data indicate an uptake of the PxB in human embryonic kidney (HEK‐293) cells, as shown by HPLC analyses of the cell lysates of cells treated with the PxB **12** 52 (Figure 2). In a mouse mutant model of the breast cancer resistance gene, evidence for transport of red phyllobilin‐type pigments (similar to RCC) in mice, as well as of protoporphyrin IX, has been provided.^53^ Further bioavailability‐ and metabolic studies will be necessary to establish a pharmacokinetic profile for specific PBs, and to determine whether the PBs are recognized and modified by metabolic enzymes in the liver.

### Phyllobilins as Antioxidants

**Figure 3:**
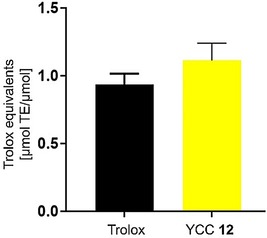
FRAP assay ^[57]^ determining the antioxidative potential of YCC **12** relative to the vitamin E derivative Trolox *in vitro*.^52^ For this assay, YCC **12** was prepared following the published protocol.31a

Secondary plant metabolites as natural food constituents are often associated with antioxidant activities that account for their health benefits (e. g. flavonoids^54^), by scavenging radicals and preventing diseases related to oxidative stress.^55^ Also for the PBs, antioxidant activities have been demonstrated *in vitro*; the phylloleucobilin (NCC) **11** and the phylloxanthobilin (YCC) **12**, for example, were shown to be potent inhibitors of lipid autoxidation.9a,^56^ Furthermore, a FRAP (ferric reducing antioxidant potential) assay revealed a strong anti‐oxidative potential for the PxB **12** (Figure 3).^52^

The FRAP assay is of particular interest, since it was used to establish a comprehensive ‘Antioxidant Food Database’, containing comparative antioxidative activity data that was collected over a period of eight years and comprises more than 3100 plant based food.^58^ Apples, for example, are listed to have an antioxidant content of 0.4 mmol/100 g. The contribution of the PleBs to this antioxidant potential of apples is yet to be determined.9a

### Structural Variety of Phyllobilins

Chlorophyll breakdown, albeit being a strictly controlled process, furnishes PBs in a large structural variety, not only due to enzymatic activities that modify the PB‐core at the ‘later’ stages (type‐I vs. type‐II PBs, formation of phyllochromobilins), but also due to late bio‐conjugations at the periphery of the tetrapyrrole core.4j,6a From a structural perspective, several ‘exotic hypermodifications’ remind of a range of compounds, ‘bioactive’ as inhibitors of cell growth28a or of high density induced apoptosis of human cancer cell lines,28b as antibacterial,^29^ antifungal,^59^ and antiviral compounds.^60^ In this respect, the banana fruit and leaves have turned out to be a particularly exciting source of hypermodified PluBs (*hm*FCCs), in which a modification of the propionic acid side chain hinders the conversion of the PluBs to its corresponding PleB and causes the fluorescent compound to accumulate in the peel and leaf. In banana peels, the total amounts of *hm*FCCs were found to correlate with the degree of ripeness of the fruit: amounts were low at the onset of ripening and peaked when the banana was ripe, followed by a decrease in the fluorescent compounds later on.

**Figure 4:**
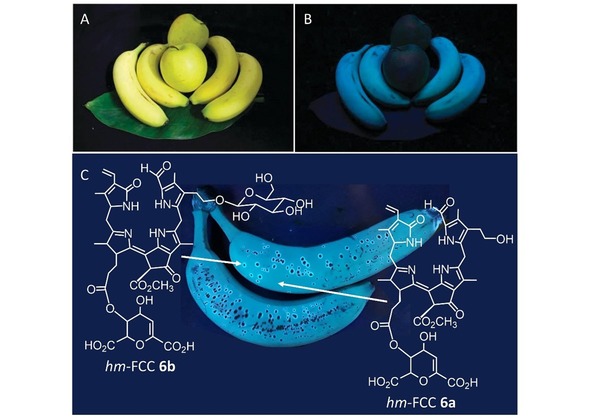
Bananas and apples, although similar in appearance in terms of color when ripe (A), look strikingly different under UV light (B), a phenomenon predominantly caused by phyllolumibilins (PluBs) in the banana peel. The peels of apples, in contrast, contain phylloleucobilins which do not luminesce. C: Differently modified PluBs are found in the peel of bananas (C). *Hm*FCC **6 b** (left) was found to accumulate in the blue luminescent zones (‘halos of cell death’ 39) surrounding senescence associated dark spots, while the more apolar *hm*FCC **6 a** was found to be the most abundant PluB on the ripe surface.6a,^39^ The bananas were photographed under UV‐light (366 nm) using a mobile phone camera.

In addition to this overall time dependency, *hm*FCC **6 b** was found to occur time dependently during later ripening stages, accumulating in ‘blue luminescent halos’ around senescent associated dark spots on the peel. The ‘late’ PluB *hm*FCC **6 b** carries an additional glucose unit compared to *hm*FCC **6 a**. The spatially restricted accumulation of *hm*FCC **6 b** in the transition zones between alive and dead cells on the banana peel is visible as blue luminescent rings that can be seen in parallel with increased amounts of *hm*FCC **6 b** (Figure 4).^39^

The physiological relevance of the blue luminescent rings and of *hm*FCC **6 b** needs yet to be resolved;^61^ anyway, the occurrence of differently ‘tagged’ phyllobilins at defined locations and during distinct windows of time indicates specific tasks of the natural products for the plant. Since the occurrence of the phyllolumibilins (PluBs) is correlated with luminescent patterns on the peel, these compounds might serve as signaling molecules, either to the outside for indicating the ripeness of the fruit, or as signal for cell death.4g,^39^,^61^ The (luminescent) PBs might also be able to protect the cell and slow down the aging process, a hypothesis that merits further investigation.

Tagging of natural products is often involved in signaling for the plant, e. g. in response to attack by herbivores, such as insects. Malonylation of secondary metabolites from *Nicotiana attenuata*, the 17‐hydroxygeranyl‐linalool diterpene glycosides (DTGs), was found to increase upon insect feeding, a response that is thought not to act as defense mechanism, since the malonyl residues were demonstrated to be removed upon ingestion in herbivores, suggesting a different role.^62^ Malonylated PBs have been identified, amongst others, in *Nicotiana rustica* 25 and *Brassica napus*.^23^ Whether the pattern of the PBs changes upon herbivore attack, and the amount of malonylated PBs increases, will be addressed in the near future and may provide a link between chlorophyll breakdown and pest infestation.

The biological investment of chlorophyll breakdown achieves de‐conjugation of the π‐system and higher water solubility of the catabolites up to the stage of the PleBs; thereafter, the typical PxBs are again more apolar and feature extended π‐systems resulting in interesting chemical and physical properties (see above), thereby partially reverting the efforts of the degradation process and intensifying the question about the biological importance of the phyllobilins.

#### On the Bioactivities of Bilins in Humans and Plants – Guidance for Future Research on the Possible Bioactivities of Phyllobilins

We discuss here the bioactivity of bilins, since structurally, phyllobilins are remarkably related to the heme‐derived bilins, the linear tetrapyrroles from breakdown of heme (see Scheme 3).4g,33b The major diagnostic structural difference between bilins and phyllobilins is the presence, in the PBs, of the additional ring E section. The 1‐formyl functionality at the macrocycle‐cleavage site is a hallmark of the type‐I PBs, is found neither in the typical bilins, nor (remarkably!) in type‐II PBs. The known bioactivities of heme breakdown products may, thus, provide basic guiding lines for the consideration of potential bioactivities of phyllobilins.

Interestingly, some degradation products of heme are known to possess a cytoprotective function; by decreasing oxidative stress, slightly elevated levels of bilirubin (BR) can aid in the prevention of a variety of diseases^63^ and are associated with longer telomeres.^64^ Also cancer related activities are known for the bile pigments; unconjugated BR was shown to induce apoptosis in colon cancer cells,^65^ and biliverdin has recently been recognized for its potential as anti‐cancer agent via inhibition of proliferation and angiogenesis.^66^ However, unconjugated BR leads to ‘neonatal jaundice’ in newborns, a serious neurological threat to babies with strong jaundice, treated by exposing them to blue light.^67^

Bilin‐type natural products from marine sources related to the phyllobilins have health benefits, too, e. g. as proposed for phycocyanobilin from algae. Phycocyanobilin was observed to inhibit NADPH oxidase activity and reduce oxidative stress;^68^ down‐regulation of NADPH oxidase has a high potential in therapeutic and preventive medicine.^69^ Furthermore, extracts of spirulina have anti‐cancer activities attributed to phycocyanin, a protein with the phycocyanobilin chromophor as prosthetic group.^70^

Heme‐derived bilins are generated in plants and play a range of important physiological roles as ‘phytobilins’.^71^ They are part of phycobilisomes, the light harvesting complexes in a range of photosynthetic organisms.^71^, ^72^ In various plants and cyanobacteria, phytobilins are also ubiquitous components of their photo‐regulatory apparatus, where they function in light receptor modules, represented, e. g., by the phytochromes.^72^, ^73^ In spite of the abundance of PBs in plants, related physiological roles that PBs could play in plants remain to be discovered.4d,4g

However, due to the extensive structural similarity between some (phyto)bilins (from heme) and phyllochromobilins (from Chl), the search for patterns of the interference of specific PBs with photo‐regulatory proteins and their biological assembly in various (lower to higher) plants^72^ might be particularly attractive targets of systematic research. Likewise, the ‘exotic’ structures of three types of PBs (**6**–**8**) as bicycloglycosidic bilin‐type compounds invite to consider a range of potential bioactivities in plants, in particular as antibacterial, antifungal, and antiviral compounds.4j,^7^

## Summary and Outlook

Chlorophyll breakdown has been regarded primarily as a detoxification process, yielding in a first instance colorless, polar metabolites. However, phyllobilins (PBs) are ubiquitous in nature and, as delineated here, their considerable diversity of structural features and patterns of biological conjugation draw remarkable connections to the bilins from heme ‘degradation’. In fact, the required biosynthetic machinery to generate the range of natural phyllobilins is considerable. Therefore, questions have arisen, such as “why would the plant invest so many resources on a mere detoxification pathway?” and “are phyllobilins not more than only degradation products?”4c,4g,6a In spite of their high abundance, their occurrence in food, and their proven in vitro anti‐oxidative activity, however, there still is a surprising lack of knowledge about their physiological roles.4d,4g

It remains to ask, what features of PBs make us expect distinctive bioactivities of PBs? Indeed, phyllobilins (PBs) entertain a strong structural relationship to the bilins from heme ‘degradation’ and occur in massive amounts in the living nature.4g They represent an array of diverse bilin‐type structures, with modifications that need metabolic energy in order to be introduced. The specific patterns of these modifications could provoke interest, e. g., into the question of the uptake of phyllobilins as part of human nutrition into the bloodstream. Hence, for cardiac glycosides, an important class of natural products that inhibit the sodium pump Na^+^/K^+^‐ATPase (in clinical use to treat heart failure and atrial arrhythmia) the varying hydroxylation patterns and (amounts and nature of) sugar units result in very different pharmacokinetic properties.^74^

The phyllochromobilins, in particular, feature a range of bio‐relevant structural and (photo)chemical properties, strikingly similar to those of their bioactive bilin‐type analogs. The occurrence of PBs with a range of bio‐relevant modifications and their existence as type‐I and type‐II PBs, offer a multitude of structure‐based opportunities. The possible correlations between the phylogenetic relationship of plant species and the pattern of ‘their PBs’, has, so far, remained unsolved,4d but is another potentially interesting issue from the pharmacological point of view. Aside of the natural PBs, as lead structures, synthetic modifications may enlarge the array of these bilin‐type compounds.^75^ With the number of structurally identified natural PBs growing steadily, correlations between botanical families and the PBs they contain may be established, and, in return, predictions about the expected PB contents of a certain plant may become possible. With regards to the potential bioactivities of PBs, such as antioxidant activity, and the occurrence of PBs in food in varying amounts, as demonstrated for apples and pears, it might be of interest to identify fruit and vegetables with a high PB contents, which may contribute to the health benefits of a plant based diet.

No doubt, it is worthwhile to look out for biological activities of the abundant but hardly explored phyllobilins^76^ and to address the probably most attractive fields of research for this task. Probably, they are physiological, pharmacological and biomedical studies targeting the metabolic roles of the PBs in humans (and higher animals), as well as plant biological and microbiological investigations concerned with potential functions of PBs in higher plants and other photosynthetic organisms, as well as their possible technological applications.

## Biographical Information

***Simone Moser** studied chemistry at the University of Innsbruck and received her PhD in 2009 under the supervision of Prof. Bernhard Kräutler, working on the structure elucidation of phyllobilins. She then pursued postdoctoral studies with Prof. Kai Johnsson at EPFL and Prof. Elizabeth M. Nolan at MIT in the fields of target identification and mode of action studies of small molecules and peptides. After one year in Analytical Development in the pharmaceutical industry (Sandoz Biopharmaceuticals, Novartis), she returned to academia and is now a group leader at the Department of Pharmacy at LMU. Her research interests lie at the interface of chemistry and biology, with the focus on the bioactivities of phyllobilins*.

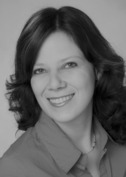

## Biographical Information

***Bernhard Kräutler** is Professor Emeritus at the Institute of Organic Chemistry and Center of Molecular Biosciences of the University of Innsbruck. His main research interests (see https://www.uibk.ac.at/organic/kraeutler/) concern the phyllobilins, vitamin B_12_ and antivitamins B_12_, functionalized fullerenes and porphyrins. Highlights of his education in chemistry were a Diploma of Chemistry at ETH Zürich (1970), a PhD in Chemistry (1977) with Prof. Albert Eschenmoser at the ETH in Zürich and postdoctoral stays with Prof. Allen J. Bard at the University of Texas at Austin (1977) and with Prof. Nicholas J. Turro at Columbia University, New York (1978). Among his honors and awards were the Werner Award of the Swiss Chemical Society (1987), the Schrödinger Award of the Austrian Academy of Sciences (2001), the Joseph‐Loschmidt Medal of the Austrian Chemical Society (2005). He is a Fellow ChemPubSoc Europe (2015), Member of the German Academy of Sciences Leopoldina (2006) and Member of the Austrian Academy of Sciences (2009)*.